# Robust phylogenetic tree-based microbiome association test using repeatedly measured data for composition bias

**DOI:** 10.1186/s12859-024-06002-2

**Published:** 2025-03-06

**Authors:** Kangjin Kim, Sungho Won

**Affiliations:** 1 Department of Applied Statistics, Gachon University, Seongnam, South Korea; 2 Institute of Health and Environment, Seoul National University, Seoul, South Korea; 3 Department of Public Health Sciences, Graduate School of Public Health, Seoul National University, Seoul, South Korea; 4 Interdisciplinary Program for Bioinformatics, College of Natural Science, Seoul National University, Seoul, South Korea

**Keywords:** Tree-based microbiome association test, Microbiota, Microbiome data, MTMAT

## Abstract

**Background:**

The effects of microbiota on the host phenotypes can differ substantially depending on their age. Longitudinally measured microbiome data allow for the detection of the age modification effect and are useful for the detection of microorganisms related to the progression of disease whose identification change over time. Moreover, longitudinal analysis facilitates the estimation of the within-subject covariate effect, is robust to the between-subject confounders, and provides better evidence for the causal relationship than cross-sectional studies. However, this method of analysis is limited by compositional bias, and few statistical methods can estimate the effect of microbiota on host diseases with repeatedly measured 16S rRNA gene data. Herein, we propose mTMAT, which is applicable to longitudinal microbiome data and is robust to compositional bias.

**Results:**

mTMAT normalized the microbial abundance and utilized the ratio of the pooled abundance for association analysis. mTMAT is based on generalized estimating equations with a robust variance estimator and can be applied to repeatedly measured microbiome data. The robustness of mTMAT against compositional bias is underscored by its utilization of abundance ratios.

**Conclusions:**

With extensive simulation studies, we showed that mTMAT is statistically relatively powerful and is robust to compositional bias. mTMAT enables detection of microbial taxa associated with host diseases using repeatedly measured 16S rRNA gene data and can provide deeper insights into bacterial pathology.

**Supplementary Information:**

The online version contains supplementary material available at 10.1186/s12859-024-06002-2.

## Background

Recent advancements in high-throughput technologies such as microarrays and next-generation sequencing have significantly elucidated the microbial world. For instance, microbiota has been found to play essential roles in the host by influencing energy homeostasis, body adiposity, blood sugar control, insulin sensitivity, hormone secretion and metabolic profiles. Moreover, ongoing research suggests potential associations between microbiota and complex diseases, including asthma, atopic dermatitis, obstructive sleep apnea, inflammatory bowel disease, and type-2 diabetes [1–6]. However, the composition of the microbiota varies from subject to subject, and the abundances of microbial taxa are often sparse with excessive zeros. Furthermore, the microbiota is affected by various factors, such as age and sex, which cause its abundance to vary considerably. Such sparsity in the data makes controlling type-1 and type-2 errors in statistical analyses difficult, and the inference of causal relationships through statistical analysis of microbiota data should be performed cautiously.

Repeatedly measured microbiota studies are useful for the detection of microorganisms related to the progression of disease and change identification over time, and provide more evidence for the causal relationship than cross-sectional studies [7]. Furthermore, the estimation of within-subject covariate effects is robust to between-subject confounders, and repeatedly measured microbiome data allow for the robust identification of microbiota effects on the risk of diseases in the host. Statistical analyses with repeatedly observed 16S rRNA gene require the adjustment of similarity among the measurements of the same subjects. However, only a limited number of methods are applicable in the repeated observation of 16S rRNA gene data, and the development of statistical methods for longitudinal studies is required for investigating the association between the human microbiome and diseases.

Xia et al. [8] comprehensively reviewed statistical methods of longitudinal data analysis in microbiome studies. These methods can be categorized into several categories: (1) standard longitudinal model, (2) overdispersed and zero-inflated longitudinal models, and (3) multivariate distance/kernel-based longitudinal models. First, the standard longitudinal model includes the linear mixed effect model (LMM) with generalized estimation equation (GEE) and generalized linear mixed effect model (GLMM) among others. LMM provides a standardized and flexible approach toward modeling both fixed and random effects. However, operational taxonomic unit (OTU) or amplicon sequence variant (ASV) abundances cannot address the sparsity issue and should be transformed or normalized to avoid the violation of distribution assumptions. Second, overdispersed and zero-inflated longitudinal models include the zero-inflated Gaussian (ZIG) mixture model, extensions of negative binomial mixed-effects (NBMM) [9], and zero-inflated negative binomial models (FZINBMM) [10]. The two-part zero-inflated beta regression model with random effects (ZIBR) extends the zero-inflated beta regression model to longitudinal data settings [11]. FZINBMM and ZIBR can analyze overdispersed and zero-inflated longitudinal metagenomics data. Finally, the multivariate distance/kernel-based longitudinal model includes the correlated sequence kernel association test (cSKAT) for continuous outcome, and the generalized linear mixed model and its data-driven adaptive test (GLMM-MiRKAT) for non-normally distributed outcome such as binary traits [12].

However, despite such developments, longitudinal microbiome data suffers from compositional bias, and only a few methods are robust to this problem. The magnitude of the sequence depth differs from subject to subject in metagenomics data, and the sums of absolute abundances for each subject are substantially different; therefore, relative abundances are generally used. However, this generates compositional bias, especially in longitudinally observed microbiome data [13], because relative abundances at each time point only provide a proportion of a taxon and it is therefore not possible to derive meaningful signals by comparing the relative abundance of the same subject over time. Furthermore, relative abundances are correlated among different taxa, and their adjustment is necessary if they were utilized as a response variable [14].

Several methods have been proposed to adjust the compositional bias. For instance, additive log-ratio (ALR) that uses a reference abundance for its denominator and centered log-ratio (CLR) that uses geometric mean can be considered. Network analysis, including sparse correlations for compositional data and sparse inverse covariance estimation for ecological association inference, can also be considered, modeling the whole community with a statistical model. However, they can only be applied to cross-sectional data, and none of them can be applied to analyzing longitudinally observed datasets [15].

In this article, we propose the phylogenetic tree-based microbiome association test for repeatedly measured data (mTMAT), which pools the abundance of OTU/ASVs based on the phylogenetic distance, thus resolving the problem of zero-inflation, making it robust to compositional bias. Through extensive simulation and real data analyses, we prove its robustness to compositional bias and its improved statistical power compared to that of other methods.

## Methods

### Phylogenetic tree

We apply similar notations and assumptions as in TMAT [16]. For this analysis, OTU/ASV profiling is performed across all subjects and time points simultaneously, with a rooted binary phylogenetic tree provided for these OTU/ASVs.

**Fig. 1:**
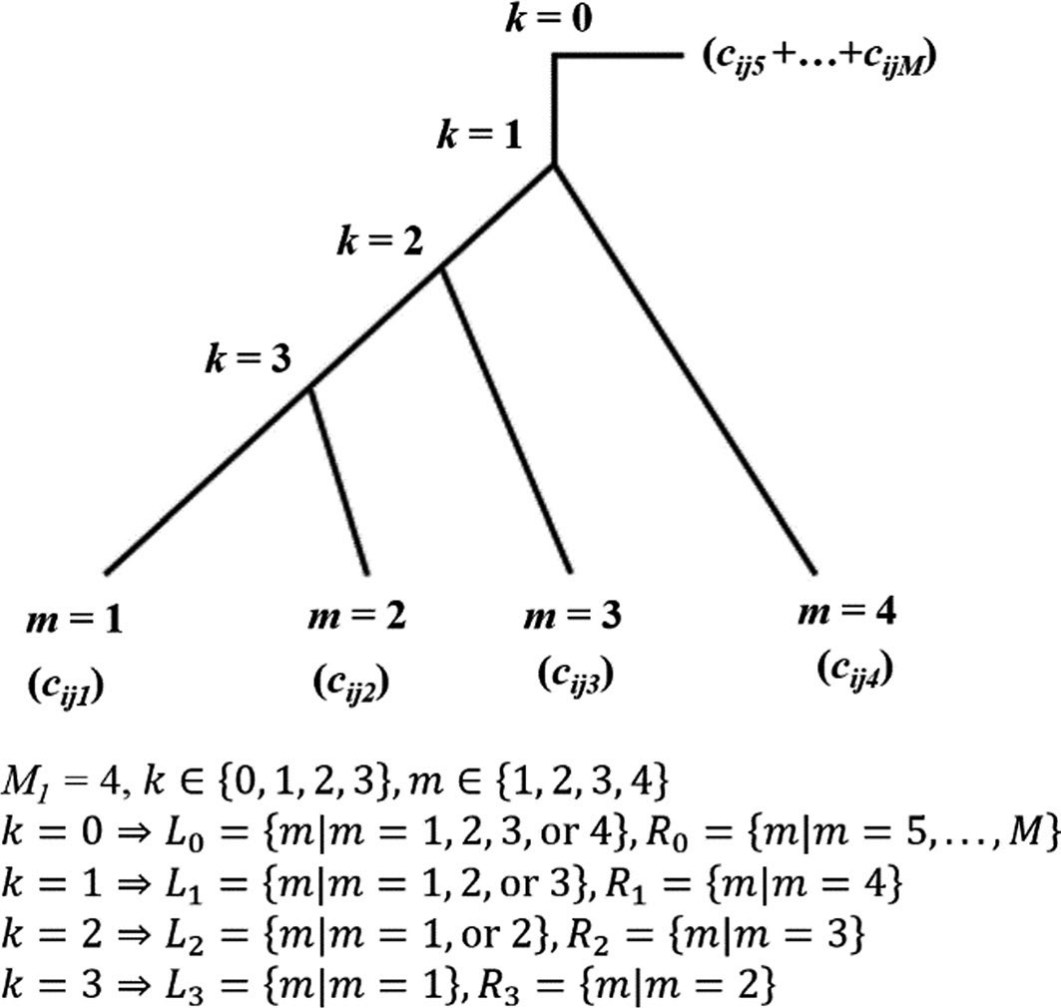
Examples of rooted binary phylogenetic trees

The first *M*_1_ OTU/ASVs are categorized under a taxonomy of interest *t*, (where *t* = 1, … *T*), for studying associations with host diseases, while the remaining *M–M*_t_ OTU/ASVs are classified into different taxonomies. Within the genus of interest, there are *M*_t_ – 1 internal nodes and *M*_1_ leaf nodes. Internal nodes are denoted by *k* (where *k* = 1, …, *M*_t_ – 1) and leaf nodes by *m* (where *m* = 1, …, *M*). Each leaf node corresponds to a single OTU/ASV. If *m* $\le$ *M*_t_, it represents a leaf node within the genus of interest, otherwise *m* belongs to a different genus. The absolute abundance of OTU/ASV *m* of subject *i* at time point *j* is denoted by ${c}_{ijm}^{(t)}$. Assuming mutations occur during transmission from an internal node k to its child nodes, the relative abundance of leaf nodes of the left child node increases for cases where the mutation occurs during transmission to the left child, and decreases if it does so during transmission to the right child node. When assessing the association between an internal node *k* and a host disease, *k* and its associated leaf nodes are considered as the test nodes and test leaf nodes, respectively. The left and right test leaf nodes correspond to the leaf nodes of the left and right child nodes of *k*, and are denoted as *L*_*k*_ and *R*_*k*_, respectively. Figure 1 provides a visual representation of theses definitions.

### Quasi-likelihood

The log-transformed counts per million (CPM) ${r}_{ijm}^{(t)}$, which is used for the edgeR package (version 3.16.5) is expressed as follows.

$${r}_{ijm}^{(t)}={log}_{2}\left(\frac{{c}_{ijm}^{(t)}+\frac{{c}_{ij\cdot }^{(t)}}{2}}{\sum_{m=1}^{M}{c}_{ijm}^{(t)}+{c}_{ij\cdot }^{(t)}}\times {10}^{6}+1\right).$$

${x}_{ij}^{(k)}$, where *k* = 1, …, ${M}_{1}-$1, is expressed as follows

$$x_{{ij}}^{{(k)}} = log\left( {\frac{{C_{{ij}}^{{(k)}} }}{{D_{{ij}}^{{(k)}} }}} \right),C_{{ij}}^{{(k)}} = \sum\limits_{{m = 1}}^{{M_{t} }} {r_{{ijm}}^{{(t)}} } \cdot I(m \in L_{k} ),D_{{ij}}^{{(k)}} = \sum\limits_{{m = 1}}^{{M_{t} }} {r_{{ijm}}^{{(t)}} } \cdot I(m \in R_{k} ).$$

Here, I(A) indicate the indicator function. As all OTU/ASVs in taxonomy *t* can be associated with the host disease, ${x}_{i}^{0}$ for such a case is expressed as follows:

$${x}_{ij}^{(0)}=log\left(\frac{{E}_{ij}^{(t)}}{{G}_{ij}}\right), {E}_{ij}^{(t)}=\sum_{m=1}^{{M}_{t}}{r}_{ijm}^{(t)}, {G}_{ij}=\sum_{m={M}_{t}+1}^{M}{r}_{ijm}^{(t)}.$$

To facilitate comprehension, we assume that${x}^{(0)}$, …, and ${x}^{({M}_{1}-1)}$ are ordered according to the sequential sequence of internal nodes commencing from the root node, as illustrated in Fig. 1. ${x}^{(0)}$ is used to test the association of all OTU/ASVs belonging to the genus of interest by pooling them, and ${x}^{(1)}$ is used for testing the root node of the phylogenetic tree. The phenotype of subject *i* at time point *j* is denoted by *y*_*ij*_ and is coded as 1 and 0 for cases and controls, respectively. To address potential arguments about how to best represent all other microbial abundances relative to the genus of interest, we considered two additional options for the value of${G}_{ij}$, both of which account for compositional bias. The first one uses ${E}_{ij}^{(t)}$ value of a reference taxon, while the second one uses the geometric mean ${\left(\prod_{t=1}^{T}{E}_{ij}^{(t)}\right)}^\frac{1}{T}$ for all taxonomies. These two options correspond to the ALR and CLR transformations, respectively. The vectors and matrices for testing the association of the genus of interest are expressed as follows:

$${\mathbf{x}}_{i}^{\left(k\right)}=\left(\begin{array}{c}{x}_{i1}^{\left(k\right)}\\ \vdots \\ {x}_{i{N}_{i}}^{\left(k\right)}\end{array}\right), {\mathbf{y}}_{i}=\left(\begin{array}{c}{y}_{i1}\\ \vdots \\ {y}_{i{N}_{i}}\end{array}\right)$$

The stacked observations across all subjects are represented as

$${\mathbf{x}}^{(k)}=\left(\begin{array}{c}{\mathbf{x}}_{1}^{\left(k\right)}\\ \vdots \\ {\mathbf{x}}_{N}^{\left(k\right)}\end{array}\right), \mathbf{X}=\left(\begin{array}{ccc}{\mathbf{x}}^{(0)}& \cdots & {\mathbf{x}}^{{(M}_{1}-1)}\end{array}\right), \mathbf{y}=\left(\begin{array}{c}{\mathbf{y}}_{1}\\ \vdots \\ {\mathbf{y}}_{N}\end{array}\right).$$

Based on such matrix notations, we provide the quasi-likelihood for repeatedly observed 16S rRNA gene data. If we denote ${R}_{i}$ and ${\sigma }_{kk}$ as a working correlation matrix and overdispersion parameter, respectively, and define ${D}_{ik}$ as a diagonal matrix with its diagonal entries being $var({x}_{ij}^{(k)})$, *j* = 1,…,${N}_{i}$, the covariance matrix for the observations of subject *i* is expressed as follows:

$${\varvec{\varSigma}}_{i}^{\left( k \right)} = \sigma_{kk} {\varvec{D}}_{ik}^{1/2} {\varvec{R}}_{i} {\varvec{D}}_{ik}^{1/2} .$$

Then, the covariance matrix ${{\varvec{\Sigma}}}^{(k)}$ can be expressed as follows:

$${\varvec{\varSigma}}^{\left( k \right)} = \left( {\begin{array}{*{20}c} {{\varvec{\varSigma}}_{1}^{\left( k \right)} } & 0 & 0 \\ 0 & \ddots & 0 \\ 0 & 0 & {{\varvec{\varSigma}}_{N}^{\left( k \right)} } \\ \end{array} } \right).$$

If we let **Z** be a design matrix for *p* covariates, including the intercept, we assume

$$E\left({{\varvec{x}}}^{(k)}|{\varvec{Z}},{\varvec{y}}\right)={\varvec{Z}}{\boldsymbol{\alpha }}_{k}+{\varvec{y}}{\beta }_{k}, var\left({{\varvec{x}}}^{(k)}|{\varvec{Z}},{\varvec{y}}\right)={\boldsymbol{\Sigma }}^{(k)}, k=0,\dots ,{M}_{1}-1.$$

Therefore, quasi-score functions for ${{\varvec{\upalpha}}}_{k}$ and ${\beta }_{k}$ can be expressed as follows:

$$U\left({\boldsymbol{\alpha }}_{k},{\beta }_{k}\right)=\left(\begin{array}{c}{{\varvec{U}}}_{\boldsymbol{\alpha }}\left({\boldsymbol{\alpha }}_{k},{\beta }_{k}\right)\\ {U}_{\beta }\left({\boldsymbol{\alpha }}_{k},{\beta }_{k}\right)\end{array}\right)=\left(\begin{array}{c}{{\varvec{Z}}}^{t}{\left({\boldsymbol{\Sigma }}^{(k)}\right)}^{-1}\left({x}^{(k)}-{\varvec{Z}}{\boldsymbol{\alpha }}_{k}-{\varvec{y}}{\beta }_{k}\right)\\ {{\varvec{y}}}^{t}{\left({\boldsymbol{\Sigma }}^{(k)}\right)}^{-1}\left({x}^{(k)}-{\varvec{Z}}{\boldsymbol{\alpha }}_{k}-{\varvec{y}}{\beta }_{k}\right)\end{array}\right).$$

Quasi-fisher information can be expressed as follows:

$${\varvec{H}}=\left(\begin{array}{cc}{{\varvec{U}}}_{\boldsymbol{\alpha }\boldsymbol{\alpha }}& {{\varvec{U}}}_{\boldsymbol{\alpha }{\varvec{\beta}}}\\ {{\varvec{U}}}_{{\varvec{\beta}}\boldsymbol{\alpha }}& {{\varvec{U}}}_{{\varvec{\beta}}{\varvec{\beta}}}\end{array}\right)=\left(\begin{array}{cc}{{\varvec{Z}}}^{t}{\left({\boldsymbol{\Sigma }}^{(k)}\right)}^{-1}{\varvec{Z}}& {{\varvec{Z}}}^{t}{\left({\boldsymbol{\Sigma }}^{(k)}\right)}^{-1}{\varvec{y}}\\ {{\varvec{y}}}^{t}{\left({\boldsymbol{\Sigma }}^{(k)}\right)}^{-1}{\varvec{Z}}& {{\varvec{y}}}^{t}{\left({\boldsymbol{\Sigma }}^{(k)}\right)}^{-1}{\varvec{y}}\end{array}\right).$$

### Score test with small sample adjustment

The null hypothesis can be expressed as follows:

$$H0: \mathbf{L}\left[\begin{array}{c}{{\varvec{\upalpha}}}_{k}\\ {\beta }_{k}\end{array}\right]=0$$

where **L** is a matrix of linear constraints with *c* rows and number of columns equal to the length of $$\left[\begin{array}{c}{{\varvec{\upalpha}}}_{k}\\ {\beta }_{k}\end{array}\right]$$ and **0** is the zero vector of matching dimension. To test the null hypothesis, *H*_0_: ${\beta }_{k}=0$, the generalized score statistics by Boos can be applied [17] by setting **L** $$=\left[\begin{array}{cc}{0}^{t}& 1\end{array}\right]$$ as follows [18]:

$${T}_{k}={{\varvec{U}}\left({\boldsymbol{\alpha }}_{k},0\right)}^{t}{\widetilde{{\varvec{H}}}}^{-1}{{\varvec{L}}}^{t}{\left({\varvec{L}}{\widetilde{{\varvec{H}}}}^{-1}\widetilde{{\varvec{B}}}{\widetilde{{\varvec{H}}}}^{-1}{{\varvec{L}}}^{t}\right)}^{-1}{\varvec{L}}{\widetilde{{\varvec{H}}}}^{-1}{\varvec{U}}\left({\boldsymbol{\alpha }}_{k},0\right)\sim {\chi }^{2}\left(df=1\right)$$

where

$$\tilde{\user2{H}} = \mathop \sum \limits_{i = 1}^{N} - {\varvec{D}}_{i}^{t} \sum_{i}^{ - 1} {\varvec{D}}_{i} ,{ }{\varvec{D}}_{i} = \left[ {\begin{array}{*{20}c} {{\varvec{Z}}_{i} } & {{\varvec{y}}_{i} } \\ \end{array} } \right],$$

$${\tilde{\mathbf{B}}} = \mathop \sum \limits_{i = 1}^{N} {\mathbf{U}}_{i} \left( {{{\varvec{\upalpha}}}_{k} ,0} \right){\mathbf{U}}_{i} \left( {{{\varvec{\upalpha}}}_{k} ,0} \right)^{t} .$$

To adjust the small sample bias, $\widetilde{\mathbf{B}}$ is further updated as follows [19]:

$${\widetilde{\mathbf{B}}}_{adj}={\sum }_{i=1}^{N}{\mathbf{D}}_{i}^{t}{\sum }_{i}^{-1}{\left({\mathbf{I}}_{i}-{\widetilde{\mathbf{P}}}_{ii}\right)}^{-1}{\mathbf{S}}_{i}{\mathbf{S}}_{i}^{t}{\left({\mathbf{I}}_{i}-{\widetilde{\mathbf{P}}}_{ii}^{t}\right)}^{-1}{\sum }_{i}^{-1}{\mathbf{D}}_{i}$$

where

$${\mathbf{S}}_{i}={\mathbf{x}}_{i}^{(k)}-\left({\mathbf{Z}}_{i}{{\varvec{\upalpha}}}_{k}+{\mathbf{y}}_{i}{\beta }_{k}\right)$$

$${\widetilde{\mathbf{P}}}_{ii}={\mathbf{D}}_{i}\left(\mathbf{I}-{\widetilde{\mathbf{H}}}^{-1}{\mathbf{L}}^{t}{\left(\mathbf{L}{\widetilde{\mathbf{H}}}^{-1}{\mathbf{L}}^{t}\right)}^{-1}\mathbf{L}\right){\widetilde{\mathbf{H}}}^{-1}{\mathbf{D}}_{i}^{t}{\sum }_{i}^{-1}$$

### Wald test with small sample adjustment

The Wald statistic with sandwich estimator with correction of small sample bias was considered [18]. ${\beta }_{k}$ can be estimated by solving the estimating equation, ${U}_{\beta }\left({{\varvec{\upalpha}}}_{k},{\beta }_{k}\right)=0,$ as follows:

$$\hat{\beta }_{k} = \left( {{\mathbf{y}}^{t} \left( {{\hat{\mathbf{\Sigma }}}^{\left( k \right)} } \right)^{ - 1} {\mathbf{y}}} \right)^{ - 1} \left( {{\mathbf{y}}^{t} \left( {{\hat{\mathbf{\Sigma }}}^{\left( k \right)} } \right)^{ - 1} \left( {{\mathbf{x}}^{\left( k \right)} - {\mathbf{Z}}{\hat{\mathbf{\alpha}}}_{k} } \right)} \right).$$

For the estimation of the variance of ${\widehat{\beta }}_{k},$ we consider the robust variance estimator with a small sample adjustment as follows

$${\widehat{V}}_{k}={\left({\sum }_{i=1}^{N}{\mathbf{y}}_{i}^{t}{\left({\widehat{{\varvec{\Sigma}}}}_{i}^{(k)}\right)}^{-1}{\mathbf{y}}_{i}\right)}^{-1}{\widehat{B}}_{adj}{\left({\sum }_{i=1}^{N}{\mathbf{y}}_{i}^{t}{\left({\widehat{{\varvec{\Sigma}}}}_{i}^{(k)}\right)}^{-1}{\mathbf{y}}_{i}\right)}^{-1}$$

where

$${\widehat{B}}_{adj}={\sum }_{i=1}^{N}{\mathbf{y}}_{i}^{t}{\left({\widehat{{\varvec{\Sigma}}}}_{i}^{(k)}\right)}^{-1}{\widehat{Cov\left({\mathbf{x}}_{i}^{(k)}\right)}}_{robust}{\left({\widehat{{\varvec{\Sigma}}}}_{i}^{(k)}\right)}^{-1}{\mathbf{y}}_{i}.$$

$${\widehat{Cov\left({\mathbf{x}}_{i}^{k}\right)}}_{robust}={\left({\mathbf{I}}_{{N}_{i}}-{\widehat{\mathbf{P}}}_{ij}\right)}^{-1}\left({\mathbf{x}}_{i}^{(k)}-{\mathbf{Z}}_{i}{\widehat{{\varvec{\upalpha}}}}_{k}\right){\left({\mathbf{x}}_{i}^{(k)}-{\mathbf{Z}}_{i}{\widehat{{\varvec{\upalpha}}}}_{k}\right)}^{t}{\left({\mathbf{I}}_{{N}_{i}}-{\widehat{\mathbf{P}}}_{ij}\right)}^{-1}$$

$${\hat{\mathbf{P}}}_{ij} = {\mathbf{y}}_{i} \left( {\mathop \sum \limits_{i = 1}^{N} {\mathbf{y}}_{i}^{t} \left( {{\hat{\mathbf{\Sigma }}}_{i}^{\left( k \right)} } \right)^{ - 1} {\mathbf{y}}_{i} } \right)^{ - 1} {\mathbf{y}}_{j}^{t} \left( {{\hat{\mathbf{\Sigma }}}_{j}^{\left( k \right)} } \right)^{ - 1} .$$

Based on this result, the robust Wald statistic of ${\beta }_{k}$ for test node *k* is expressed as follows:

$$T_{k,wald} = \hat{\beta }_{k}^{t} \left( {\hat{V}_{{\mathbf{k}}} } \right)^{ - 1} \hat{\beta }_{k} = \frac{{\hat{\beta }_{k}^{2} }}{{\hat{V}_{{\mathbf{k}}} }}\sim { }\chi^{2} \left( {df = 1} \right){ }\,under\,{ }H_{0} .$$

### mTMAT

Following the approach described in Kim et al. (2020), we combined statistics to test the null hypothesis ${H}_{0}:{\beta }_{0}={\beta }_{1}=\dots ={\beta }_{{M}_{1}-1}=0$ using the minimum p-value method. If p-values for *T*_*k*_ are denoted by *pT*_*k*_, the proposed mTMAT_M_ statistics are defined as:

$${mTMAT}_{M}=min\left\{\begin{array}{ccc}p{T}_{0},& \cdots & , p{T}_{{M}_{1}-1}\end{array}\right\}.$$

The p-values $$\begin{array}{ccc}p{T}_{0},& \cdots & , p{T}_{{M}_{1}-1}\end{array}$$ are asymptotically independent, as shown in previous studies [16]. Therefore, under ${H}_{0}$ the distribution of mTMAT_M_ follows a beta distribution with parameters (1, M_1_).

If the sample size is small, the normality of *T*_*k*_ under *H*_0_ may not be achieved, and the assumption of the quasi-score test can be violated. If we apply the inverse normal transformation to ${x}_{ij}^{(k)}$, then the same statistics can be obtained. This is denoted by ${T}_{k}^{INT}$. Rank-based inverse normal transformation with an adjustment parameter of 0.5 was used for the transformation, and data with tie values were mapped to the same value in the transformed data [20]. Then, mTMAT_IM_ is expressed as follows:

$$mTMA{T}_{IM}=min\left\{\begin{array}{ccc}p{T}_{0}^{INT},& \cdots & , p{T}_{{M}_{1}-1}^{INT}\end{array}\right\}\sim beta\left(1,{M}_{1}\right) under {H}_{0}.$$

### KARE cohort data

The Korea Association REsource (KARE) cohort is a prospective study cohort involving subjects from the rural community of Ansung and the urban community of Ansan in South Korea. It began in 2001 as part of the Korean Genome Epidemiology study [21]; we used data from 2,072 urine samples from 691 participants in 2013, 2015, and 2017. Their 16S rRNA gene amplicon sequencing data used in the study were obtained from the NCBI Sequence Read Archive database under project accession number PRJNA716550 [22]. For paired-end sequencing of the V3-V4 region of the bacterial 16S rRNA gene, the widely used primers 16S_V3_F (5’- TCGTCGGCAGCGTCAGATGTGTATAAGAGACAG-CCTACGGGNGGCWGCAG-3’) and 16S_V4_R (5’-GTCTCGTGGGCTCGGAGATGTGTATAAG-AGACAGGACTACHVGGGTATCTAATCC-3’) were used. Adaptor sequences were detected and removed using the CUTADAPT software (version 4.4) with a minimum overlap of 11 bp, maximum error rate of 10%, and a minimum length of 10 bp [23]. Sequences were merged using CASPER (version 0.8.2) with a mismatch ratio of 0.27 and filtered by the Phred (Q) score, resulting in sequences of 350–550 bp in length [24, 25]. After the merged sequences were dereplicated, chimeric sequences were detected and removed using VSEARCH (version 2.3.4) and the Silva Gold reference database for chimeras [26]. The open-reference Operational Taxonomic Unit (OTU) picking was based on the EzTaxon database using UCLUST (version 1.2.22) with a 97% sequence identity threshold [27, 28]. The phylogenetic trees based on EzTaxon database were obtained through the SINA method [29] using the reference sequences available from the EzTaxon database. We calculated the proportion of each species among the total taxa and determined the mean value across all subjects. If the resulting value was < 0.001, the species was excluded [30]. A histogram of the read count distribution is shown in Additional file 1: Fig. S1. Among the 691 subjects, those with a read count of < 3,000 or for whom genomic data were not available in any phase were excluded. As a result, 1179 samples from 393 subjects, including 70 genera, were used for the simulation analysis.

### Simulation studies

We conducted extensive simulations to evaluate the performance of mTMAT with two types of datasets. One dataset with 393 subjects who participated in all three phases from KARE cohort and the generative dataset based on microbiomeDASim [31]. For the KARE cohort dataset, we defined disease status based on a creatinine level threshold of 1.15, with values above 1.15 as cases and others as controls; however, this specific threshold was not critical, as the focus was on maintaining an appropriate case–control ratio. We followed a methodology similar to that described in Kim et al. (2020) to evaluate the effect of disease status permutation on the identification of causal taxa, which in this context refers to taxa designated as associated with disease within the simulation framework. The case-to-control ratios were assumed to be 1:1 or 1:3 depending on the simulation scenario, and for the 1:3 ratio, cases were rounded down, and controls were rounded up to maintain the total sample size. Briefly, the disease status of subjects was permuted, and a single test node was randomly selected from the internal nodes of the phylogenetic tree. From the leaf nodes descending from this test node, causal taxa were chosen randomly at three different levels: a single taxon, 50% of the taxa, or 90% of the taxa, corresponding to p = 1 taxon, 50%, and 90%, respectively. Notably, when p = 1, indicating a single taxon associated with the disease, the phylogenetic structure does not offer additional information for mTMAT.

For the simulation, the sample variances of ${c}_{ijm}^{(t)}$ for causal taxa were denoted by ${\widehat{\sigma }}_{mm}^{(t)}$. For affected subjects, an increment $\delta =\beta {\widehat{\sigma }}_{mm}^{(t)}$ was added to the observed absolute abundances of the selected causal taxa, where $\beta$ values were set to 0, 0.01, 0.02, or 0.04. The absolute abundances of non-causal taxa remained unchanged. The case with $\beta =0$ was used to estimate empirical type-1 error rates, while non-zero $\beta$ values were employed for statistical power estimation. Type-1 error rates were assessed at significance levels of 0.1, 0.05, 0.01, and 0.005 using 5,000 replicates, while empirical power was evaluated at the 0.05 significance level with 500 replicates. Unless described otherwise, these replicates were used throughout the study. Type-2 error, defined as 1 − power, was therefore indirectly addressed through the power analysis.

For comparison with mTMAT_M_ and mTMAT_IM_, GLMM-MiRKAT (version 1.2), FZINBMM (version 1.0), linear mixed model (LMM) with arcsine square root transformation (LMM-arcsine), and LMM with log transformation (LMM-log) with nlme package (version 3.1) were considered. Additionally, phylogenetic tree-based microbiome association test (TMAT, version 1.01), optimal microbiome regression-based kernel association test (oMiRKAT, version 0.02), adaptive microbiome-based sum of powered score (aMiSPU, version 1.0), and the Wilcoxon test were included to compare how cross-sectional methods perform in the context of repeatedly measured data. This allows for benchmarking mTMAT_M_ and mTMAT_IM_ against methods that do not explicitly account for temporal correlations, providing insights into the importance of considering longitudinal structures in microbiome association studies. Association analyses were conducted at the genus level. FZINBMM, LMM models, and Wilcoxon were applied by pooling all species within each genus. Each genus consisted of multiple species, and oMiRKAT and aMiSPU were applied to species belonging to each genus.

For mTMAT_M_ and mTMAT_IM_, robust wald and score statistics with four different choices of working correlation matrix: identity, compound symmetry (CS), first-order autoregressive (AR1) and unstructured (UN), were considered. For aMiSPU and oMiRKAT, we followed the same parameters to Kim et al. (2020) using 500 and 5,000 permuted replicates to assess power and type-1 error rates, respectively. For GLMM-MiRKAT, Unifrac distance which is default choice is used for its distance metrics. Unifrac distance requires observed read counts, and therefore, samples with zero read counts were excluded from the analyses involving GLMM-MiRKAT, oMiRKAT, and aMiSPU. Additionally, these methods cannot handle genera consisting of a single taxon, so such cases were omitted from the statistical power analyses for these genera.

Following the simulation with the generated dataset with microbiomeDASim, Identity, CS, and AR1 with different parameter values are assumed for the simulation, and type-1 error estimates were compared for different uses of working correlation matrices for mTMAT_IM_. The mean value of relative abundance and proportion of zero count samples were estimated from the KARE cohort study for all the genera; the genera with first quantile, median, and third quantile sparsity level were selected for the simulation. The values were 52%, 64%, and 73%.

We also evaluate the robustness of the proposed method to the compositional bias. Different choices of ${G}_{ij}$ corresponding to ALR and CLR are also considered. The KARE dataset was simulated 2000 times with simulation parameters *N* and the ratios of the cases and controls were 50 and 1:3, respectively. Then a genus containing more than one species was selected and assumed to be associated with a phenotype with $\beta =0.15$ and *p* = 50%. Additionally, a species outside the selected genus was randomly chosen and assumed to be associated with the same phenotype using the same β value. To evaluate how changes in the abundance of this external taxon influence the bias estimate for the selected genus, its abundance was increased by its standard deviation multiplied by factors of 0, 1, 5, 10, 50, and 100. Then the mean and interquartile range of bias estimate of the selected genus was calculated and compared with a different value of the multiplier.

### Pregnant microbiome data

We used publicly available datasets from Romero [32], who conducted a retrospective case–control longitudinal study that included non-pregnant women (n = 32) and pregnant women who delivered at term (38 to 42 weeks) without complications (n = 22) using pplacer and version 0.2 of the vaginal community 16S rRNA gene database [33] for the taxonomic classification. The neighbor joining method based on the Bray–Curtis dissimilarity index was used to obtain a phylogenetic tree [34]. The pregnant dataset includes data on the race, days after the first visit (GDColl), household income, maternal education, and baby gender.

## Results

### Results from simulated data

The performances of mTMAT_M_ and mTMAT_IM_ were evaluated using simulated data. Additional file 1: Fig. S2 shows the overall distribution of microbial composition. Results for mTMAT_M_ are presented in Additional file 1: Table S1, which indicates that inflation of type-1 error rates occurred as the number of case samples and total samples increased. Conversely, mTMAT_IM_ adequately preserved the nominal type-1 error with a slight inflation when unstructured correlation (Table 1 and Additional file 1: Table S2).

**Table 1:** Type-1 error estimates of mTMAT_IM_ and other statistical methods from repeatedly measured microbiome data at three time points at the significance levels 0.1, 0.05

Method	Working correlation matrix	α = 0.1						α = 0.05					
		Case: Control = 1: 1			Case: Control = 1: 3			Case: Control = 1: 1			Case: Control = 1: 3		
		N = 30	N = 50	N = 100	N = 30	N = 50	N = 100	N = 30	N = 50	N = 100	N = 30	N = 50	N = 100
mTMAT_IM_	Identity	0.0884	0.0963	0.0933	0.0999	0.0921	0.1041	0.0413	0.0493	0.0451	0.0463	0.0458	0.0495
mTMAT_IM_	CS	0.0882	0.0957	0.0915	0.1004	0.0938	0.1039	0.0421	0.0503	0.0454	0.0446	0.0451	0.0498
mTMAT_IM_	AR1	0.0893	0.0959	0.0923	0.0990	0.0943	0.1036	0.0413	0.0499	0.0454	0.0460	0.0449	0.0498
mTMAT_IM_	UN	0.0901	0.0954	0.0905	0.1003	0.0941	0.1039	0.0397	0.0478	0.0447	0.0476	0.0453	0.0499
GLMM-MiRKAT		0.1320	0.1377	0.1301	0.1450	0.1466	0.1347	0.0914	0.0889	0.0875	0.0922	0.0974	0.0846
FZINBMM		0.4882	0.4548	0.4770	0.4778	0.4625	0.4411	0.4209	0.3915	0.4162	0.4055	0.3908	0.3786
LMM-arcsin		0.1094	0.1328	0.1482	0.0926	0.0982	0.0994	0.0537	0.0681	0.0804	0.0449	0.0497	0.0467
LMM-log		0.1021	0.1115	0.1223	0.0922	0.0934	0.0905	0.0466	0.0529	0.0613	0.0421	0.0475	0.0436
TMAT_IM_		0.0988	0.1085	0.1235	0.1342	0.0927	0.0929	0.0489	0.0580	0.0660	0.0613	0.0448	0.0456
TMAT_M_		0.0978	0.1132	0.1289	0.1336	0.1042	0.0944	0.0463	0.0564	0.0642	0.0660	0.0505	0.0448
Wilcoxon		0.1034	0.1174	0.1337	0.1320	0.0947	0.0938	0.0496	0.0558	0.0635	0.0631	0.0488	0.0449
oMiRKAT		0.1041	0.1141	0.1298	0.1308	0.0950	0.0945	0.0509	0.0514	0.0585	0.0678	0.0527	0.0436
aMiSPU		0.0839	0.0973	0.1108	0.1056	0.0800	0.0691	0.0435	0.0471	0.0536	0.0568	0.0426	0.0335

The ratios between cases and controls were assumed to be 1:1 and 1:3. The total sample size is denoted by N, and we considered N = 30, 50, and 100. For a 1:3 ratio, cases were rounded down and controls were rounded up to maintain the total sample size. All subjects were selected without replacement. Type-1 error estimates were calculated with 5,000 replicates at the significance levels 0.1, 0.05

GLMM-MiRKAT, FZINBMM and LMM models are designed to be used as longitudinal microbiome data and can be compared with mTMAT_IM_ and mTMAT_M_. FZINBMM and GLMM-MiRKAT could not preserve type-1 error rates with extremely high type-1 error estimates for FZINBMM. GLMM-MiRKAT suffered singular matrix problem during calculating the test statistics (Table 1). In this case, the resulting p-values were excluded for the estimation of type-1 error rate and power.

We also evaluated the effect of the number of leaf nodes (Additional file 1: Table S3), and the results showed that mTMAT_M_ became slightly conservative when the number of leaf nodes exceeded 5, but that mTMAT_IM_ was less affected. The result with more than 15 leaf nodes can be dependent on specific genera chosen with a small number of genera.

Additional file 1: Table S6 evaluated the effect of sparsity on the type-1 error rate using an approach same to that described in Kim et al. (2020). The results indicate that the type-1 error rates for FZINBMM were the most inflated, with some inflation also observed for GLMM-MiRKAT when mean sparsity exceeded 20%. Although GLMM-MiRKAT employs permutation-based p-values, which are generally robust to non-normality, their validity can be affected by heteroscedasticity. A high degree of sparsity may introduce heteroscedasticity, contributing to the observed type-1 error inflation. While there was some inflation noted for mTMAT_M_, the type-1 error rates for mTMAT_IM_ were well-controlled.

The effect of the assumed correlation matrix for different scenarios was evaluated with the use of microbiomeDASim [31]. Identity, CS, and AR1 with different parameter values are assumed for the simulation with different uses of working correlation matrix, robust score statistic and for mTMAT_IM_ (Additional file 1: Table S4). The result shows that mTMAT_IM_ preserved type-1 error for all the scenarios.

To further assess the type-1 error performance of mTMAT_IM_ with larger sample sizes, we conducted additional simulations for *N* = 100, 200 and 400. These simulations confirmed that type-1 error rates remain well-controlled and do not exhibit inflation as *N* increases (Additional file 1: Table S5).

**Fig. 2:**
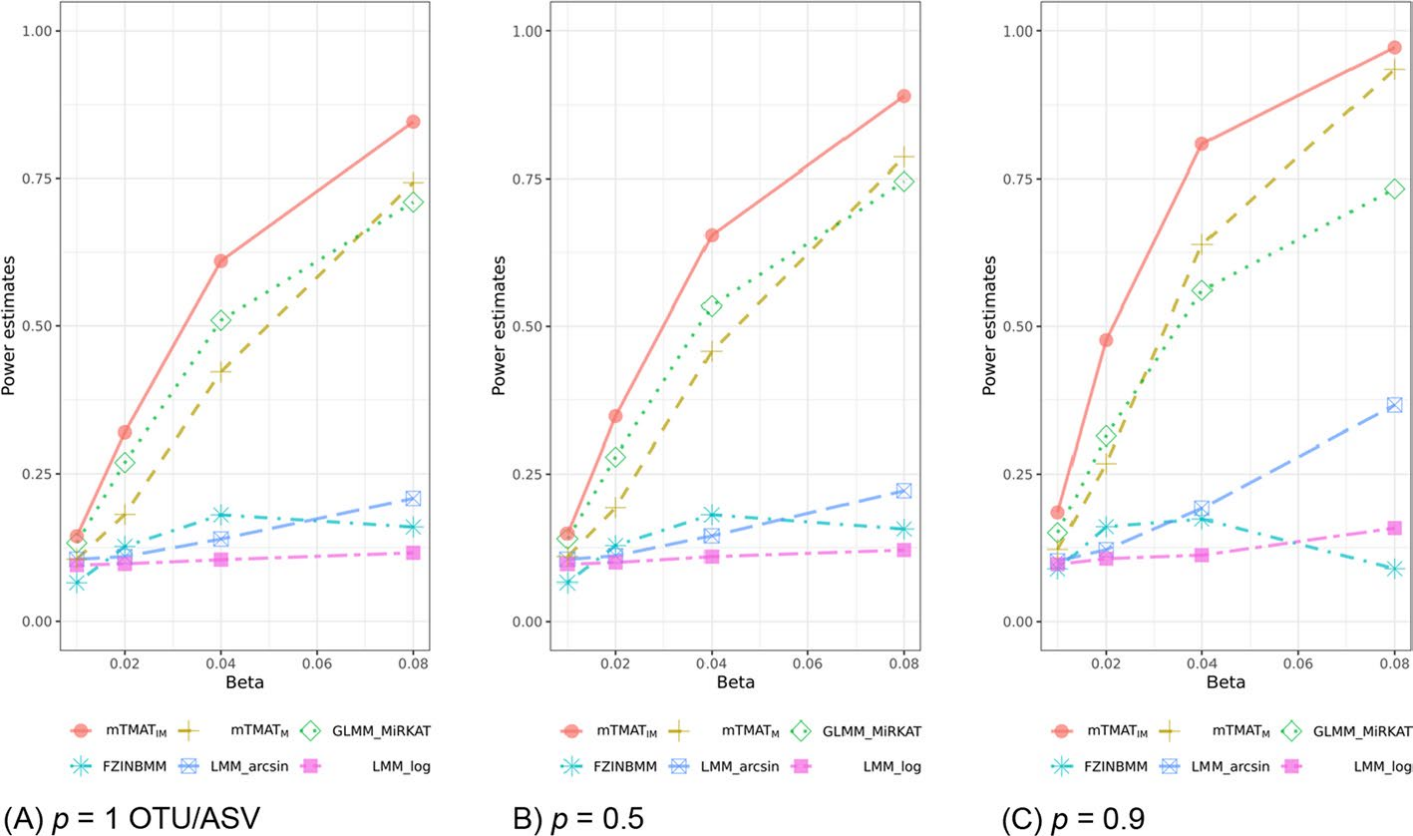
Power estimates for genera consisting of more than one taxon. Power estimates at the significance level 0.05 were calculated with 500 replicates. We generated simulation data based on read counts from datasets and considered genera with more than one taxon. For statistical methods whose type-1 errors are violated, their P-values were adjusted with the simulated data, which makes all statistical methods preserve the nominal significance level. We assumed that the total sample size (N) is equal to 50, the ratio of cases and controls is set to be 1:3. Identity working correlation matrix and robust score statistic were used for mTMAT

We also calculated statistical power estimates with 2,000 replicates at the 0.05 significance level and compared them with those of other statistical methods. The significance levels for each method were adjusted based on the statistics from the simulation to calculate type-1 error for a valid performance comparison. The threshold is determined as the percentiles of the p-values calculated in the type-1 error simulation under null hypothesis. We considered genera comprising two or more taxa. In Fig. 2, mTMAT_IM_ usually outperformed the other methods. The performance of GLMM-MiRKAT was comparable with mTMAT_M_. FZINBMM and LMM-log exhibited significantly less power than other methods.

Additional file 1 Fig. S3 shows the results of genera comprising one or more taxa. GLMM-MiRKAT can only be calculated if more than one taxon is available. Therefore, it was excluded from this comparison. mTMAT_M_ and mTMAT_IM_ can be applied in such scenarios, and the results showed that the proposed method was the most efficient.

Comparison with methods for cross-sectional analysis (Additional file 1: Fig. S4) shows that TMAT_M_, TMAT_IM_, and mTMAT_IM_ showed high statistical power. aMiSPU had the highest power estimate when beta was 0.02.

As shown in Additional file 1: Fig. S5, we evaluated the statistical power in relation to the number of leaf nodes. Keeping the number of causal taxa constant, we observed that the statistical power estimates decreased as the number of leaf nodes increased. Optimal performance was observed for mTMAT_IM_; however, GLMM-MiRKAT exhibited a high level of type-1 error rate with the leaf node ranging between 6 and 15. The sparsity levels were also considered, and statistical power estimates were compared. Power estimates were maximized at the middle-group sparsity level (Additional file 1: Fig. S6). The type-1 error and the power of group and time and interaction effect are also estimated using simulation dataset with microbiomeDASim package. The type-1 error rates for the group, time (Additional file 1: Table S7), and interaction effects (Additional file 1: Table S8) are well controlled for mTMAT_IM_ and mTMAT_M_, while LMM-arcsine and LMM-log exhibit type-1 error inflation. We verified that mTMAT_IM_ and mTMAT_M_ can successfully capture group and time effects with the other included as a covariate. Both methods also detected interaction effects with adequate control of type-1 error rates. Although LMM-arcsine and LMM-log showed higher power than mTMAT_IM_ and mTMAT_M_ in this simulation scenario, their reliability is limited due to the observed type-1 error inflation.

Additional file 1: Fig. S7 shows the effect of compositional bias. mTMAT_IM_ and FZINBMM had a smaller bias than other methods when the level of compositional bias was high. All three types of mTMAT_IM_ had smaller interquartile ranges of bias than FZINBMM, indicating that mTMAT_IM_ successfully mitigated compositional bias compared to other methods. Furthermore, the default *G*_*ij*_ option for mTMAT_IM_ consistently outperformed the ALR and CLR options in terms of reducing both bias and variability, highlighting its robustness in addressing compositional bias in the dataset.

In summary, we confirmed that mTMAT_IM_ is generally the most efficient method among those available in our simulations. mTMAT_IM_ considers phylogenetic tree structures, uses log CPM transformation, correction of compositional bias by taking a proportion among taxa, and considers correlations among repeatedly measured samples, which makes it superior to other methods. The overall power comparison results for cross-sectional methods are consistent among previous studies on TMAT [16]; however, the type-1 error rate for TMAT was inflated with longitudinal microbiome data. GLMM-MiRKAT is the second most powerful but failed to preserve type-1 rates and cannot be applied in analysis with a single taxon. Furthermore,

GLMM-MiRKAT is based on oMiRKAT, and they both used the kernel method and permutation approaches, which can be very computationally intensive, especially if the sample size increases [16].

### Real data analysis

The pregnant datasets were analyzed with mTMAT, GLMM-MiRKAT, FZINBMM, and LMM with the arcsine square root transformation and LMM with log transformation to explore the association between microbiota and the pregnancy status of the women. The pregnant dataset includes the race, days after the first visit (GDColl), household income, maternal education, and gender of baby. The overall composition is presented in Additional file 1: Fig. S8; the overall composition change was clear after > 300 days. Additional file 1: Fig. S9 shows that the change can be related to the pregnancy state. PERMANOVA analysis result shows the associated phenotype that explained microbiome variability. Race was the covariate with the least p-value of 0.06 (Additional file 1: Fig. S10). Table 2 shows that mTMAT_IM_ found 11 significant genera. FZINBMM, LMM-arcsine, and LMM-log found 16, 14, and 14 significant genera, respectively. As shown in the simulation study, most of the detected genera as significant only by FZINBMM can be false positives.

**Table 2:** Association analysis results of the Pregnant dataset

Family taxonomy	Genus taxonomy	mTMAT_IM_	mTMAT_M_	GLMM-MiRKAT	FZINBMM	LMM arcsine	LMM log
*Lactobacillaceae*	*Lactobacillus*	0.01984	0.01549	0.00416	0.00000	0.00000	0.00000
*Tissierellaceae*	*Anaerococcus*	0.02110	0.02275	0.01247	0.00000	0.00000	0.00000
*Tissierellaceae*	*Peptoniphilus*	0.02599	0.02110	0.00416	0.00000	0.00000	0.00000
*Veillonellaceae*	*Dialister*	0.01984	0.02110	0.06983	0.00000	0.00000	0.00000
*Campylobacteraceae*	*Campylobacter*	0.01984	0.02275	NA	0.00000	0.00000	0.00000
*Tissierellaceae*	*Finegoldia*	0.04797	0.04647	NA	0.00000	0.00000	0.00001
*Porphyromonadaceae*	*Porphyromonas*	0.13031	0.10538	NA	0.00000	0.00000	0.00000
*Streptococcaceae*	*Streptococcus*	0.01984	0.02275	NA	0.00000	0.00000	0.00000
*Actinomycetaceae*	*Varibaculum*	0.55456	0.34057	NA	0.00001	0.28831	0.93412
*Prevotellaceae*	*Prevotella*	0.01984	0.02110	NA	0.00000	0.00000	0.00000
*Tissierellaceae*	*1–68*	0.84255	0.67380	NA	0.00006	0.00165	0.00171
*Lactobacillaceae*	*WAL_1855D*	0.09401	0.05409	NA	0.00000	0.00000	0.00000
*Tissierellaceae*	*Mobil uncus*	0.01984	0.02110	NA	0.00000	0.00000	0.00000
*Tissierellaceae*	*Atopobium*	0.01984	0.02522	NA	0.00000	0.00000	0.00000
*Bifidobacteriaceae*	*Gardnerella*	0.99925	0.79316	NA	0.00014	0.53031	0.59088
*Actinomycetaceae*	*Actinomyces*	0.01984	0.02110	NA	0.00000	0.00000	0.00000

GLMM-MiRKAT requires more than one taxon for calculation; therefore, genera with a single taxon show ‘NA’ for this method

mTMAT_IM_ shared most of the significant genera with other methods. The most significant genera was *Lactobacillus*, which is consistent with the findings of the original study [32]. Additional file 1: Fig. S11 shows a Venn diagram comparing the numbers of significant genera implicated by the various applied methods. As LMM-arcsine and LMM-log differ only in their transformation, the methods shared all 16 detected genera. FZINBMM detected two more genera that were not detected by other methods. mTMAT_IM_ shared all the 12 detected genera with other methods. Results for genera significantly associated with at least one method at the FDR-adjusted 0.05 significance level are summarized.

**Fig. 3:**
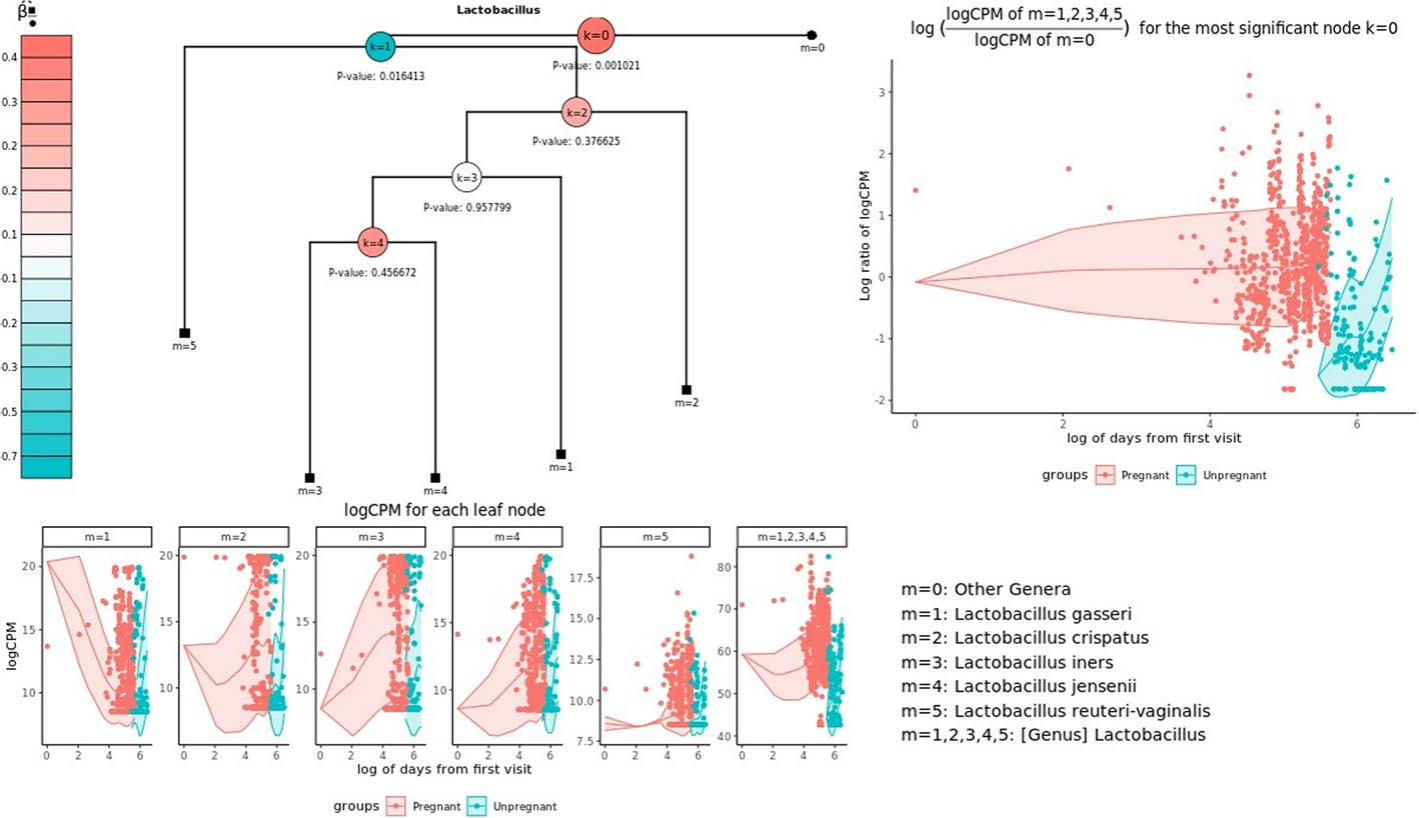
Taxon distributions of significantly associated genus *Lactobacillus*. Relative proportions of taxa belonging to *Lactobacillus* at different time points were plotted. Each taxon has its corresponding leaf node; leaf nodes in black square and black circle indicate that they are in L_k_ and R_k_    , respectively. $\hat{\beta }_{{{\text{black}}\;{\text{square/black}}\;{\text{circle}}}}$ indicates the mean difference of $\log (C_{ij}^{(k)} /D_{ij}^{(k)} )$ between pregnant and unpregnant subjects after adjusting for covariates, and the red internal node indicates that taxa in the left test leaf nodes are more abundant in pregnant subjects. The most significant node is enlarged

Figure 3 shows the distribution of taxa under *Lactobacillus*. Lactobacillus has five leaf nodes and the relative abundances of all the leaf node m = 1, 2, 3, 4 and 5 were higher in the pregnant group. *Lactobacillus* was observed to be more abundant in the pregnant group than in the control group. This finding aligns with existing research indicating that *Lactobacillus* species are more abundant in pregnant women. Notably, a deficiency in vaginal *Lactobacillus* species has been linked to an increased risk of preterm delivery [32, 35]. Additional file 1: Fig. S12, 13, 14, 15, 16, 17 and 18 showed the taxon distributions of other associated genera. These results confirm that the genera identified using mTMAT may be associated with delivery. Therefore, mTMAT successfully detected associated genera.

## Discussion

The importance of microbiome-host interactions has been known for more than a century [36], and the occurrence of many human diseases has been found to be related to bacterial communities.

Microbiome data has phylogenetic structure and certain unique properties, including high dimensionality, rarity, and heterogeneity beyond composition. These properties cause multiple statistical problems when analyzing data across microbial composition and integrating multi-omics data such as large p and small n, dependencies, over-dispersion and zero inflation. The classical correlation and related methods throughout the microbial association study were applied in the actual study and used in the development of new methods. However, owing to those problems related to metagenomic analysis, traditional approaches are infrequently utilized for more complex models, such as longitudinal models including linear mixed models and generalized linear mixed models. Furthermore, those methods do not properly address compositional bias and may lead to erroneous results owing to limitations in relative abundance data [37].

In this study, we propose a novel approach, mTMAT, for identifying taxa associated with host diseases. This method is based on quasi-scores for internal nodes in a phylogenetic tree. It can account for various correlation structures and provides robust estimation for mis-specified correlation structures while maintaining statistical validity with small sample sizes and high statistical power. By leveraging log CPM transformation, integrating abundances, and taking ratios between integrated abundances based on the phylogenetic tree, mTMAT not only reduces sparsity issues and compositional bias but also provides insights into species-level associations by assessing patterns across closely related species.

This property is achieved by using log CPM transformation and integrating abundances based on the phylogenetic tree. Compositional bias correction is accomplished by taking ratios between two sets of integrated abundances. mTMAT leverages the phylogenetic tree structure, allowing us to incorporate the relationships between microbial species. This tree-based approach not only reduces sparsity issues but also provides insights into species-level associations by assessing patterns across closely related species.

The final statistic is formed by combining these quasi-scores to obtain a single p-value, which allows mTMAT to effectively detect taxa associated with disease status. Importantly, due to the independence of the statistical scores at internal nodes, the minimal p-value can be calculated directly (Kim et al., 2020). Comparative analyses against other methods like GLMM-MiRKAT, LMM-arcsine, LMM-log, and FZINBMM across various simulations demonstrate that mTMAT not only maintains the correct nominal type-1 error rate but also exhibits superior statistical power. Moreover, it is computationally more efficient than permutation-based methods such as GLMM-MiRKAT. While LEfSe is a useful tool for detecting differential abundance in certain microbiome datasets, it takes a different approach to statistical assessment, which may limit its ability to rigorously assess statistical significance.

However, despite its usefulness, mTMAT has several limitations. First, mTMAT combines the statistics for each internal node, and multiple comparisons occur when the number of leaf nodes is large. Second, we evaluated the performance of the proposed methods with extensive simulations, but this result cannot be generalized. Third, the choice of database, OTU clustering methods or use of ASV is likely to affect the statistical property of mTMAT. In our previous research, we showed that the effect of a mis-specified phylogenetic tree is generally not substantial. However, further investigation is necessary, which we will undertake in our follow-up research.

## Conclusions

mTMAT can help researchers easily perform fast and effective microbiome-wide association analysis, thereby comprehensively elucidating the interaction mechanism of the entire microbiota with the human body.

## Supplementary Information

Additional file 1.

## Data Availability

The 16S rRNA amplicon sequencing metagenomics datasets for Korea Association REsource cohort are accessible from the NCBI Sequence Read Archive database under project accession number PRJNA716550. mTMAT was implemented as an R package. Detailed information is available at https://healthstat.snu.ac.kr/software/mtmat.
